# Novel fabrication of hydrophobic/oleophilic human hair fiber for efficient oil/water separation through one-pot dip-coating synthesis route

**DOI:** 10.1038/s41598-022-11511-2

**Published:** 2022-05-10

**Authors:** Yang Chenxi, Wang Jian, Zhang Haiou, Cao Tingting, Zhou Hang, Wang Jiawei, Bai Bo

**Affiliations:** 1 Institute of Land Engineering and Technology, Shaanxi Provincial Land Engineering Construction Group Co., Ltd., Xi’an, 710075 China; 2 ShaanXi Provincial Land Engineering Construction Group Co., Ltd., Xi’an, 710075 China; 3 Key Laboratory of Degraded and Unused Land Consolidation Engineering, The Ministry of Natural Resources, Ltd., Xi’an, 710075 China; 4 Shaanxi Provincial Land Consolidation Engineering Technology Research Center, Xi’an, 710075 China; 5 Key Laboratory of Subsurface Hydrology and Ecological Effects in Arid Region of the Ministry of Education, Chang’an University, No. 126 Yanta Road, Xi’an, 710054 Shanxi China; 6 School of Water and Environment, Chang’an University, Xi’an, 710054 China

**Keywords:** Biomaterials, Nanoscale materials

## Abstract

Frequent oil spill accidents and industrial wastewater discharge has always been one of the most severe worldwide environmental problems. To cope with this problem, many fluorine-containing and high-cost materials with superwettability have been extensively applied for oil–water separation, which hinders its large-scale application. In this work, a novel human hair fiber (HHF)-polymerized octadecylsiloxane (PODS) fiber was fabricated with a facile one-pot dip-coating synthesis approach, inspired by the self-assembly performance and hydrophobicity of OTS modification. The benefits of prominent hydrophobic/lipophilic behavior lie in the low surface energy, and a rough PODS coating was rationally adhered on the surface of HHF. Driven solely by gravity and capillary force, the HHF-PODS showed excellent oil/water separation efficiency (> 99.0%) for a wide range of heavy and light oil/water mixtures. In addition, HHF-PODS demonstrated durability toward different harsh environments like alkaline, acid, and salty solutions.

## Introduction

In recent years, the frequent occurrence of discharge of industrial oily wastewater and oil spills has long been focused on the risk to environmental protection and human health^1–4^. To solve these oil-leakage problems and protect the limited water resources, many advanced materials^5,6^, equipment^7^ and technologies for sustainable and efficient water purification have gained rapidly increasing research interests^8,9^. With the rapid development of materials and interfacial science over the past decade, absorption and separation strategies become the most promising strategy for the removal of oil contaminations owing to high efficiency, low cost, and environmentally friendly advantages^10,11^. What’s more, absorption and separation strategies can transform oil into a solid or semisolid phase for further utilization^12^. Benefiting from these merits, preparation advanced absorption and separation materials are of great significance to solve the problem of oil spills and discharge of industrial oily wastewater. In general, adsorption and separation materials require intriguing wettability, i.e., hydrophobic/lipophilic or hydrophilic/oleophobic^13^. Specifically, these special wettable materials can be used to absorb or separate oil–water mixture according to its special affinity for liquid. From this point of view, the development of advanced oil/water separation materials with environmental compatibility, high hydrophobicity, low cost, good reusability, and efficiency are significant for the problems of oil spills and discharge of industrial oily wastewater.

Recently, there have been many reports showing the potential to prepare hydrophobic/oleophilic functional materials for oil–water separation^14–16^. For instance, Oscar et al.^17^ successfully fabricated the functionalized three-dimensional graphene sponges from graphene oxide solutions with different concentrations and trichloro(1H,1H,2H,2H-perfluorooctyl) silane. These advanced 3D graphene sponges showed excellent absorption efficiency of hexane solution. Eunjoo et al.^18^ fabricated a membrane using polyamide 6 (PA6) as the substrate, and trichloro(1H,1H,2H,2H-perfluorooctyl) silane (F-POSS) was used to modify the substrate of PA6 with a fluorinated silane (F-silane) to endow a primary hydrophobic coating on the surface of PA6. This obtained membrane showed good separation and purification performance. However, it has been verified that these absorption and separation materials still have limitations for commercialization due to the fluorine in the molecules^19^. Although the fluorine family has been extensively utilized as oil/water separation and absorption materials for decades, environmental and health risks have recently attracted attention due to its persistent and bioaccumulative characteristics^20,21^. Moreover, these special wettability materials involve complicated chemical/instrumentation reaction processes, high cost of the substrate, toxic chemicals, and environmental incompatibility. Therefore, new oil/water separation materials with facile fabrication processes, good environmental compatibility, good reusability, and fluorine-free properties are significant for the development of the advanced oil/water separation materials.

Human hair fiber (HHF), an important solid waste, mainly covalently linked by 18-methyldocosanoic acid (18-MEA) and its protein components^22,23^. Notably, the tightly cross-linked outer layers provide good stability and a strategy of protection against mechanical collision, which meet the complex environmental challenge. Moreover, water can easily pass through the endocuticle, the low cross-link the density of endocuticle combined with its hydrophilic state makes it highly water swellable^23^. Relying on these effects, HHF is a kind of natural nanocomposite absorption substrate with good economic performance. Moreover, HHF, as a natural polymer, will not have a negative effect on water when it is used as an absorbent and oil/water separation material^24^. Herein HHF, a cheap solid waste, was rationally selected as a substrate to fabricate the advanced oil/water separation material by attaching hydrophobic coating onto the HHF surface. The core of this innovation lies in the selection of HHF as the substrate, and the special wettability of the rough hydrophobic coating is endowed by a simple dip-coating and self-assembly method, thereby promoting oil/water separation capacity. Moreover, the as-obtained modified HHF could be conducted to selectively absorb several oils or organic solvents while repelling water completely. The HHF-PODS separate oils of different densities by a simple homemade oil/water mixture separation testing system, and all separation efficiencies were higher than 99.0% for different density oils/water mixtures. Overall, the results of this study provide a measure to prepare hydrophobic/oleophilic solid waste for application in the oil/water separation field.

## Experimental

### Material

Human hair fiber (HHF) was obtained from a local barber shop (Shaanxi, China) (The length is 5–20 cm, the diameter is 70–80 μm). Engine oil and castor oil were obtained from a local shop (Shaanxi, China). Ethanol and methylbenzene were supplied by Tianjin Chemical Reagent Factory (Tianjin, China). Octadecyltrichlorosilane (OTS) was furnished by Aladdin (China). All chemicals were used directly.

### Preparation of HHF-PODS

The raw HHF was cleaned in ethanol and deionized water and dried in an oven at 60 °C. Then, OTS-toluene solution was prepared by adding 0.5 mL OTS into 20 mL toluene solution. The HHFs were immersed in OTS-toluene solution for 15 min. Then, the HHF was removed and dried (humidity 50%, temperature 15 °C). The dried HHF were cured in an oven at ambient temperature to obtain the modified HHF-PODS.

### Characterization

The functional groups were confirmed using a PerkinElmer FTIR System 2000 via KBr pellet. The morphology of HHF and HHF-PODS was carried out with field-emission scanning electron microscopy (Hitachi S-4800, Japan) using an In-Lens detector operated at accelerating voltages of 5 and 20 kV. Elemental analysis was performed using an energy dispersive spectroscopy (EDS) detector equipped with an SEM. Contact angles were observed by a Krüss CCA200 contact angle goniometer at ambient temperature and the volumes of probing liquids in the measurements were approximately 5.0 μL. The values were averages from goniometers at least three different positions for HHF and HHF-PODS.

### Measurements of oil absorption and oil/water separation capacity

To elucidate the maximal oil absorption capacity of hydrophobic/oleophilic HHF-PODS for different oils, absorption tests of oil absorption were carried out in toluene, petroleum ether, machine oil, castor oil, and the final maximal absorption capacity was calculated by an average value of 3 times.

1 $$Q = \frac{{\text{G}}_{2}-\left({G}_{0}+{G}_{1}\right)}{{G}_{1}},$$

where *Q* (g/g) represents the oil absorbency, defined as grams of oil per gram of the modified HHF-PODS, *G*_1_ (g) and *G*_2_ (g) are the weights of samples before and after oil absorption, and *G*_0_ (g) are the weights of absorption bag.

The HHF-PODS was placed at the bifurcation and completely covered the hole. Then, the oil/water mixture (1:1, V/V) was poured into the top of the container, and the oil and water were driven by gravity and capillarity. The oil in the oil–water mixture and the separated oil are *V*_0_ and *V*_1_, respectively. The separation efficiency is defined as *Q* (%) and the separation efficiency is calculated using the following formula:

2 $$Q =\frac{{V}_{1}}{{V}_{0}} \times 100\%.$$

The flux of the immiscible oil/water mixtures separation procedure was elucidated by Flux = *V*/S*t*, where *V* is the volume of the permeated oil (mL), S is the contact area (cm^2^) of HHF-PODS, and *t* is the separation time (min)^25^.

## Results and discussion

### Formation of hydrophobic/lipophilic HHF-PODS

**Scheme 1:**
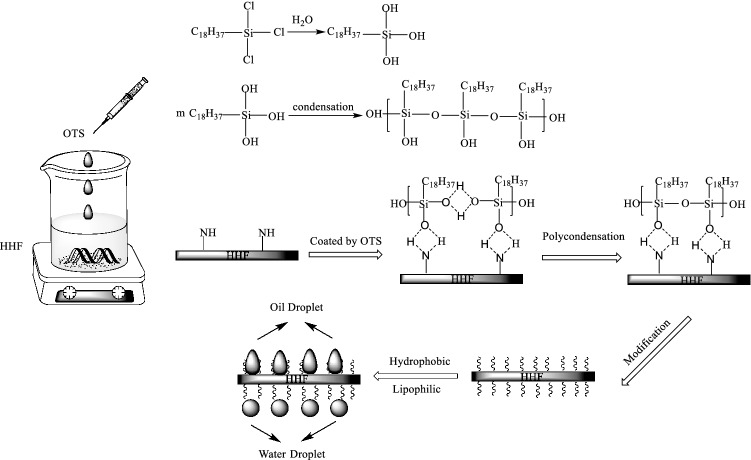
Schematic illustration of the formation mechanism of the PODS and modified human hair fiber.

HHF-PODS was prepared by the self-assembly of PODS onto the HHF skeleton through a facile dip-coating method. The proposed synthesis procedure and reaction mechanism are elucidated in the Scheme 1.

On the whole, the formation of a PODS hydrophobic coating consists of a two-step process: hydrolysis and condensation^26,27^. First, OTS was injected into the toluene solution. Trace amounts of water in toluene and water in air hydrolyze the OTS to generate free –OH groups. Specifically, the water required for the hydrolysis of OTS to form free –OH groups generally come from the solution and air. In consequence, humidity is a significant factor for OTS hydrolysis^28^. McGovern^29^ and Lieberman^30^ showed that the optimum amount of water required to prepare a densely packed monolayer was between that of the spiked solvent (0.3 mg/100 mL) and that of the anhydrous substrate (approximately 0 mg/100 mL). From this point of view, the 0.03% water content of analytical pure toluene can fully hydrolyze OTS and the influence of humidity on the formation of the PODS self-assembled film is negligible. Second, the OTS after hydrolysis is cross-linked by intermolecular polycondensation of –Si-OH groups on neighboring molecules, forming PODS in the later high-coverage stages^31,32^. Third, when the hydrolyzed OTS physisorbed on the substrate surface, some PODS molecules organized themselves into crystalline or near crystalline order due to different temperatures, and the formation characteristics of coating depended on the deposition temperature^33^. Relying on these effects, the PODS coating will be highly ordered and tightly packed at low temperatures (T < 16 °C). Therefore, the lower 15 °C was chosen as the reaction temperature in this experiment.

Predicting the structure and composition of the obtained HHF-PODS, this material might be very suitable for the preparation of superior absorbent and oil/water separation materials for oil spillage. First, the HHF substrate has good absorption ability for liquids due to the low cross-link density of endocuticles and high swellability. Moreover, central to the functional groups and structure of proteins and amino acids, which exhibit both inertness and selective reactivity, provide brand-newed insight into the chemical modifications of HHF. Moreover, HHF, as a natural polymer waste, will not have a negative effect on water when it is used as an oil/water absorbent. Second, the HHF substrate after curing at ambient temperature yielded a hydrophobic PODS nanocoating, and the prominent hydrophobic/lipophilic behavior from the low surface energy and rough PODS coating was rationally adhered on the surface of the HHF. Such unique hydrophobicity inevitably provides advantages for oil/water separation. From these points of view, the formed HHF-PODS composite materials, combined with the synergistically high absorptivity of HHF and the separation ability of hydrophobicity/lipophilicity, may shine new light for solving industrial oily wastewater, oil spills and waste utilization.

**Figure 1:**
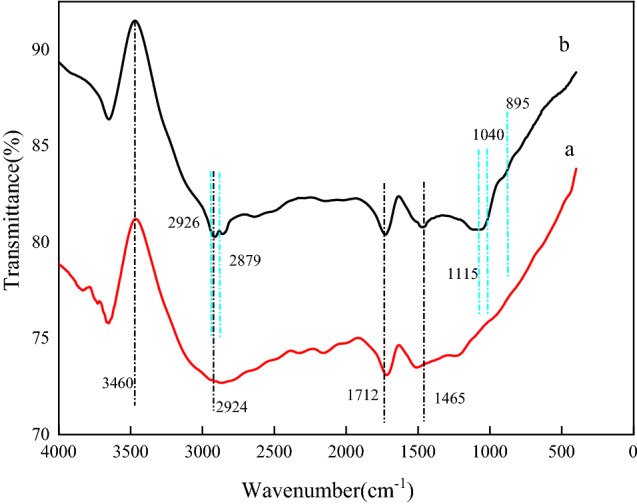
FTIR spectra analysis of HHF (a) and HHF-PODS (b).

To validate the successful synthesis of HHF-PODS, the FTIR spectra of HHF and HHF-PODS were registered. The FTIR results are filed in Fig. 1. In Fig. 1a, the spectrum of HHF exhibited prominent peaks at 3460 cm^−1^, which were assigned to the stretching vibrations of –OH and –NH. The intramolecular hydrogen bonds of amino acids and the hydrogen bonds formed with hydroxyl groups cause such strong absorption peaks. Peaks at 2924 cm^−1^ were indicative of C–H bending of the methylene group. What’s more, two small peaks at 1712 cm^−1^ and 1465 cm^−1^ were attributed to stretching vibrations of C=O and –NH bonds in amino acids^34^. The above peaks were characteristic absorption peaks in HHF. Moreover, compared with the FTIR spectrum of HHF (Fig. 1a), HHF-PODS (Fig. 1b) had new characteristic absorption peaks after dip-coating modification. The peaks at approximately 2926 and 2879 cm^−1^ may be attributed to the typical stretching vibration of –CH_2_ in the PODS coating^35^. Moreover, the HHF-PODS has weak characteristic absorption peaks at 1115 cm and 1040 cm^−1^, which are attributed to the Si–O–Si asymmetric stretching mode confirming the formation of long chain linear polysiloxane^36^. The peaks at 895 cm^−1^ were attributed to the incomplete polycondensation of ODS. Relying on the weak peak at 895 cm^−1^, it can be verified that almost all ODS molecule cross-linked by Si–O–Si condensation with each other, demonstrating the existence of PODS within the structure of HHF-PODS^37^. Ultimately, it can be concluded that the PODS self-assembled layer successfully adhered to the surface of the HHF.

**Figure 2:**
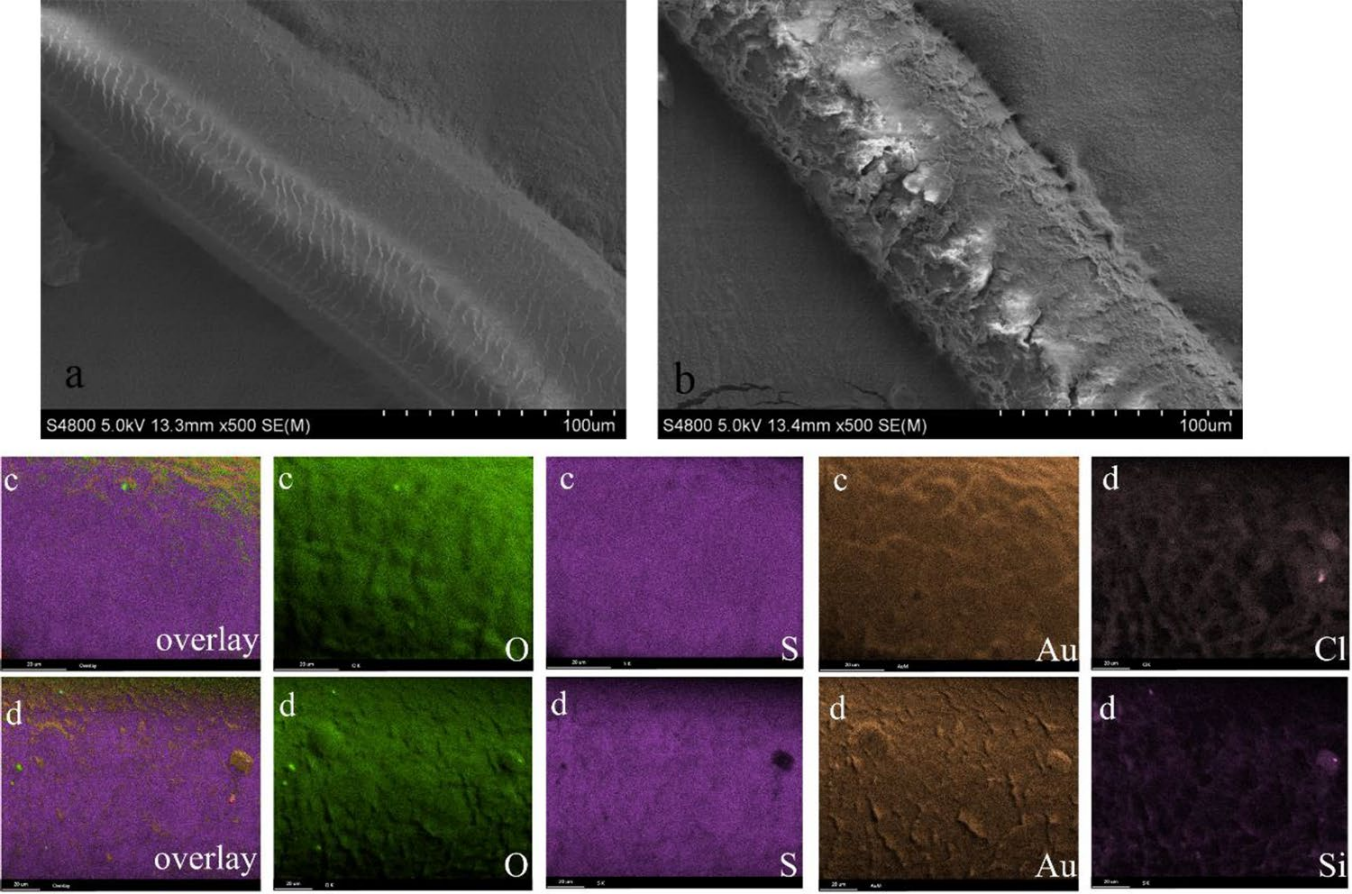
SEM photos of HHF (**a**) and HHF-PODS (**b**). Elemental mapping images of HHF (O, S, Au) (**c**) and HHF-PODS (O, S, Au, Si, Cl) (**d**).

The surface characteristics of the obtained materials were further registered in SEM images. Figure 2 shows the surface morphology of the HHF (a) and HHF-PODS (b).

Figure 2a shows an intrinsic HHF surface structure, which offered highly accessible rough structure. Specifically, the hair cells are fused densely and oriented parallel to the axis of the hair fiber. Each hair cell is packed with fine, axially oriented filaments (microfibrils and macrofibrils)^23,38,39^. The different packing dispositions of macrofibrils make the surface of the hair rough^23^. However, HHF surface is still smooth in large scale. Consequently, this smooth structure endows HHF with good hydrophilicity. Unlike the relatively smooth surface of HHF, HHF-PODS showed a rough surface in the image. Such morphology variation of HHF-PODS provides solid evidence that the PODS were successfully adhered onto the HHF surface.

Figure 2c,d shows the corresponding mapping images of the HHF and HHF-PODS, respectively. From Fig. 2c, an overlay of element EDX maps verified the clear distribution of the elements. The surface EDS mapping of HHF shows a large amount of O and S elements, which is determined by the composition of HHF. Furthermore, Au on the surface of the HHF was introduced by ion sputtering before SEM characterization. After modification with OTS, the Si signal remained strongly enhanced. Moreover, Cl on the surface of HHF-PODS is that some OTS are not completely hydrolyzed, which leads to the introduction of Cl into the surface of HHF-PODS. Elemental mapping analysis verified that we successfully synthesized the HHF-PODS by dip-coating.

### Wettability of materials

The wetting behavior of material surfaces is dependent on the chemical composition and the morphological structure of surfaces. In general, the Young formula was used to elucidate the wettability of the smooth surface^40,41^:

3 $$\mathrm{cos }\theta = \frac{{\upsigma }_{\mathrm{sv}}-{\upsigma }_{\mathrm{sl}}}{{\upsigma }_{\mathrm{lv}}},$$

where $\sigma$_sv_, $\sigma$_lv,_ and $\sigma$_sl_ are the interfacial tensions of the solid–vapor, liquid–vapor, and solid–liquid phases, respectively. Nevertheless, the general separation material surface is not absolutely smooth. Therefore, the Wenzel formula was used to determine the contact angle w of liquids on a relatively rough surface by Eq. (4):

4 $$ {\text{cos}}\theta_{{\text{w}}} = r{\text{cos}}\theta , $$

where *r* represents the roughness coefficient, which is the ratio of the actual area of a rough surface to the geometrically projected area, and *r* is usually greater than 1.

When water does not impregnate the rough surface, the formation of air pockets between solid–liquid interface transpires, which further enhances the contact angle of the hierarchical surface, and then the contact angle of such a surface is given by the Cassie–Baxter equation^42^:

5 $$ {\text{cos}}\theta_{{\text{w}}} = rf{\text{cos}}\theta - {1} + f, $$

where *f* is defined as the fraction of the solid surface contacting the different liquids. The reflections from air bubbles trapped under the water droplet validate the Cassie-Baxter wetting state^36,43^ (Fig. S2).

**Figure 3:**
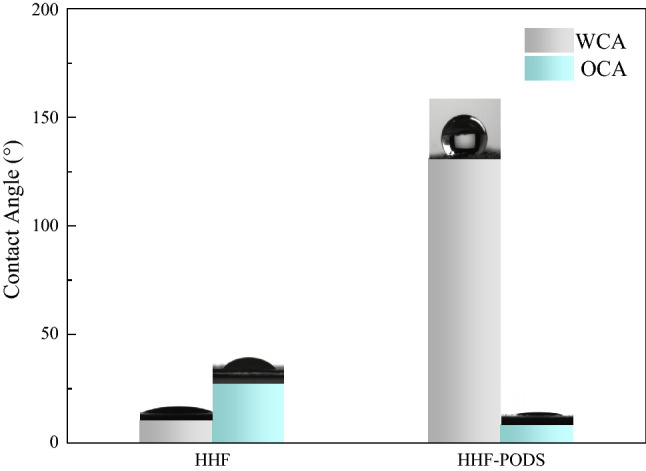
The different contact angles of water and oil on the HHF and HHF-PODS surfaces.

Specifically, the hydrophobicity can be improved through the introduction of the microstructures^44,45^. This phenomenon may be attributed to the introduction of air pockets between the solid surface and the liquid, thus improving the hydrophobicity^46^. Moreover, a suitable rough structure can synergistically turn a relatively smooth oleophilic solid surface into a more oleophilic surface^41^. Hereby, studying the wetting behavior of the sample is very significant for the oil/water separation problem. In this study, the wettability of HHF and HHF-PODS was filed in Fig. 3 by measuring the contact angle on the obtained material surface. As depicted in Fig. 3, the WCA and OCA of HHF are 10° and 27°, respectively, which indicate that HHF is an amphiphilic substrate. This result also verifies that HHF has unique properties; that is, low cross-linking density combined with its hydrophilic behavior makes it highly water swellable. Obviously, the WCA and OCA of HHF-PODS are 132° and 8°, respectively, indicating that the self-assembled hydrophobic coating was rationally attached to the surface of HHF. Meanwhile, this wetting behavior verifies the significance of surface roughness and low surface energy coating for the fabrication of hydrophobic/oleophilic materials.

### Oil absorption capacity

**Figure 4:**
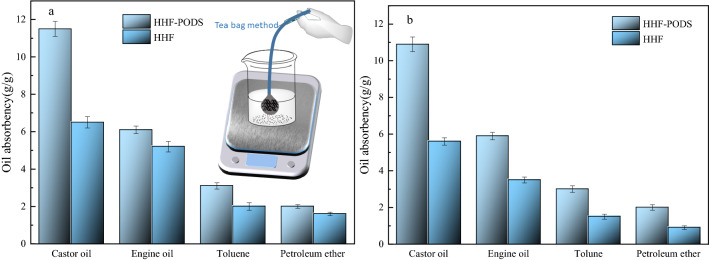
Maximum absorption capacity of HHF and HHF-PODS for different oils in the pure oil system (**a**) and the oils floating on the water surface system (**b**).

The absorption capacities of the raw HHF and as-prepared HHF-PODS for different organic solvents and oil/water mixtures are shown in Fig. 4. In Fig. 4a, the absorption capacities of HHF-PODS for castor oil, motor oil, toluene, and petroleum ether are 11.5, 6.1, 3.1, and 2.0 g/g, respectively. Nevertheless, the absorption capacity of HHF is only 6.5, 5.2, 2.0, and 1.6 g/g. Obviously, this shows that the modification of HHF improves the oil absorption ability of the HHF substrate. This phenomenon indicates that the as-prepared HHF-PODS improves the oil absorption capacity. In theory, the reason for this phenomenon is that HHF-PODS synergistically reduces the surface energy and increases the surface roughness of the HHF. In consequence, the modification improves the oil affinity. Additionally, HHF and HHF-PODS have the largest absorption capacity for castor oil. The possible reason for this phenomenon was that the high viscosity oil can adhere to the substrate surface more easily. Sticking to this principle, HHF and HHF-PODS showed good absorption properties for castor oil.

In view of practical applications, the ability to absorb oil in oil/water mixtures is an important indicator for evaluating the performance of adsorbents. To study the oil absorption performance of the as-obtained materials in oil–water mixtures, HHF and HHF-PODS were put into the oil–water mixture to recover the oil, and the results are exhibited in Fig. 4b. Figure 4b shows that the maximum absorption capacity of HHF-PODS for castor oil, motor oil, toluene, and petroleum ether is 10.9, 5.9, 3.0, and 2.0, respectively. Comparing the results with the absorption capacity in organic solvents, the oil absorption has a slight decrease. As anticipated, the possible reason for the slight decrease in oil absorption capacity is that water occupies the surface absorption site of HHF.

### Application in water/oil separation

**Figure 5:**
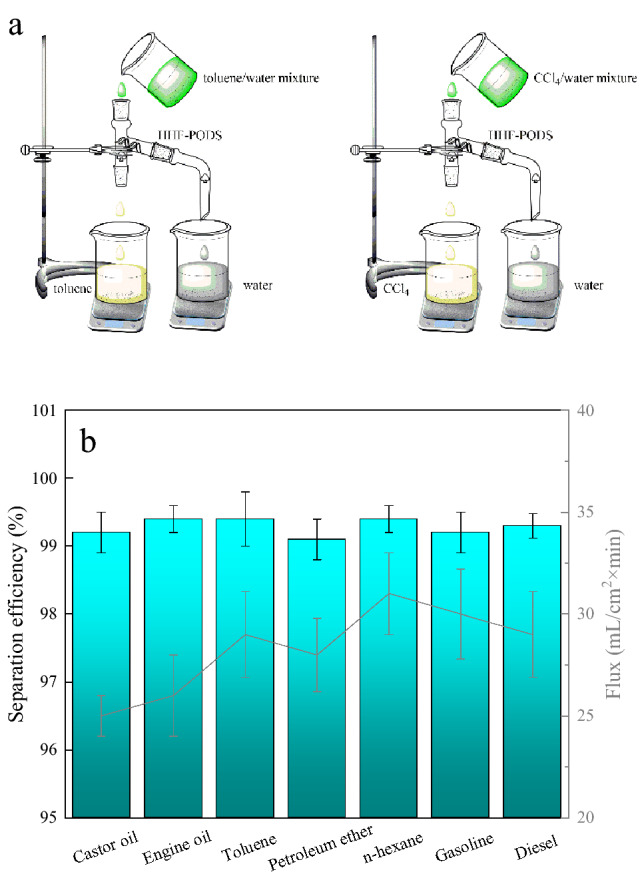
(**a**) Schematic diagram of the homemade oil/water mixture separation system. (**b**) The separation efficiency and flux of HHF-PODS for different organic solvents/water mixtures.

The separation of oil from an oil/water mixture was conducted by putting hydrophobic/oleophilic HHF-PODS into an oil/water mixture. To intuitively verify the oil/water mixture ability of the as-prepared HHF-PODS, a homemade oil/water mixture separation testing system was carried out to assess the oil/water mixture separation performance of the as-obtained HHF-PODS, and the oil–water separation schematic diagram is shown in Fig. 5a. The oil/water mixture is poured into a tube. The oil spread on the HHF due to the capillary effect and drain vertically through the HHF into the beaker by gravity. Meanwhile, the water droplets are spherical on the surface of HHF-PODS due to the hydrophobicity of HHF-PODS and then flow horizontally through the tube into another beaker. Simultaneously, the homemade oil–water separation system can be conducted continuously, which verifies that HHF-PODS maintains hydrophobicity/lipophilicity even when wetted by organic solvents^47^. From Fig. 5b, the quantitative separation efficiency was also studied, and can be calculated by the ratio between the weight of oil initially added to the oil/water mixture and after separation. All oil/water separation efficiencies were higher than 99.0% through calculation.

As HHF-PODS is compactable in continuous oil water separation experiments, the fluxes were tested after compacting the HHF-PODS until stabilization. The various immiscible oil/water mixtures were used to assess the oil water separation capacity as shown in Fig. 5b. The different oil fluxes of HHF-PODS are around 30 mL/cm^2^ × min driven by gravity. Moreover, the HHF-PODS exhibited good separation efficiency without external pressure. It is easy to know that the HHF-PODS can separate oil and water quickly and effectively with high flux. From Table 1, the oil–water separation performances of HHF-PODS is comparable or even superior to the materials in reference. This oil water separation performance owing to the wettability of HHF-PODS.

**Table 1:** Comparison of the wettability and oil–water separation performances for the HHF-PODS with the sample in references.

Samples	Wettability	Oil water separation efficiency (%)	Refs.
Waste brick grain (WBG)	Underoil WCA: 138.3°	99.1	^48^
Potato residue coated-mesh (PRCM)	WCA > 150° Underwater with OCA: 152° ± 1.3°	96.5	^49^
Waste from pulp modified by silane	WCA: 156°	99	^50^
Porous waste epoxy resin (PEP)	WCA: 120°	99.99	^51^

### Durability of the HHF-PODS toward different environments

**Figure 6:**
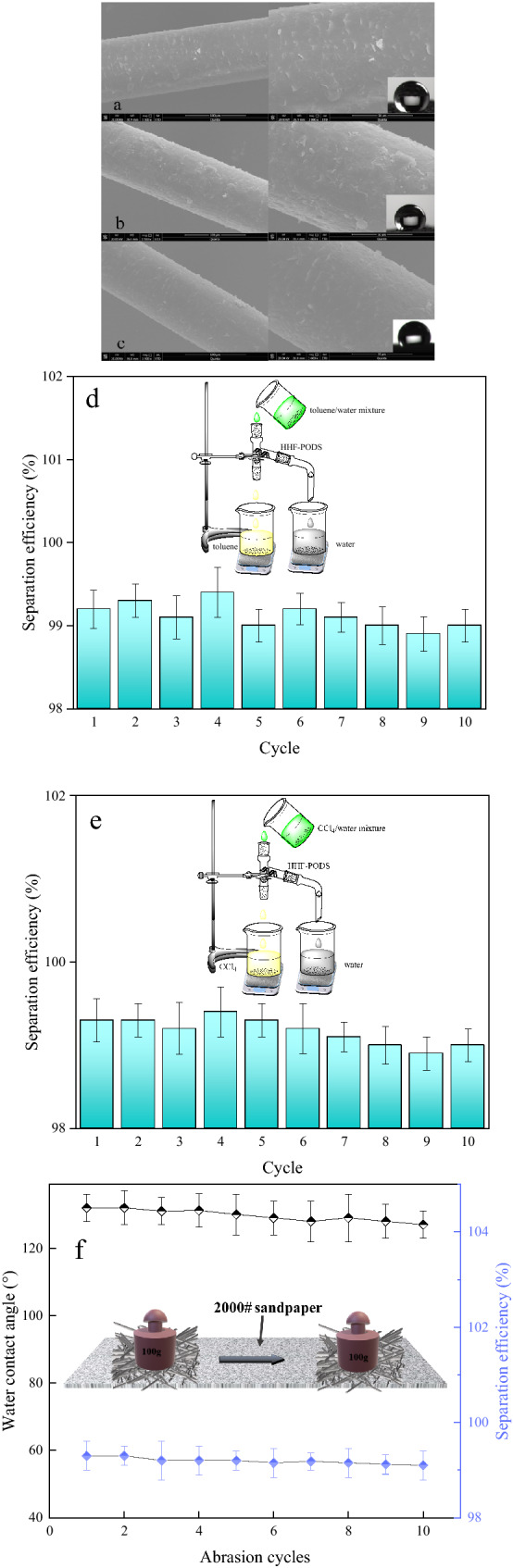
After immersion in pH 2, 7 (3.5 wt% NaCl solution) and 10 for 4 h, the morphology of HHF-PODS was almost unchanged, and a dense coating of PODS on the HHF-PODS surface was preserved, while intact fibrosis also verified the excellent resistance. (**a–c**) Reusability experiments of HHF-PODS for separating oil/water mixtures. The light oil (toluene)/water mixture was separated 10 times (**d**). The heavy oil (CCl_4_)/water mixture was separated 10 times (**e**), with an oil/water mixture separation efficiency higher than 99.0%. The WCA of HHF-PODS and separation ability after the 10 successive reusability tests with sand paper (**f**).

In general, the stability of the obtained material surface wettability toward different environments is valuable for investigating in addition to the oil/water mixture separation efficiency^52,53^. To further verify the durability of the HHF-PODS toward different harsh environments, such as alkaline, acidic, and salt solutions. The obtained HHF-PODS was immersed into the abovementioned solutions for 4 h, and the microtopography and WCAs of the HHF-PODS are shown in Fig. 6 (pH 2: 123°, Fig. 6a; 3.5 wt% NaCl: 131°, Fig. 6b; pH 10: 121°, Fig. 6c, respectively). Interestingly, although the WCA of the as-prepared HHF-PODS under acidic and alkaline conditions was less than that at 3.5 wt% NaCl (neutral pH), it still shows hydrophobicity (greater than 120°). Moreover, the microtopography remained almost constant. This phenomenon could infer that during the harsh environmental process, the rough PODS coating firmly adhered to the HHF surface. Consequently, the HHF-PODS showed excellent resistance to extreme conditions.

Furthermore, the efficiency of separating the light oil (toluene)/water mixture and heavy oil (CCl_4_)/water was still satisfactory after 10 cycles. The efficiencies were all higher than 99.0% for both toluene/water mixtures and CCl_4_/water mixtures. These separation results are well in line with the SEM analysis and wetting behavior and can help better understand the oil/water separation and durability of HHF-PODS. In consequence, this HHF-PODS can withstand harsh environments, maintain separation capacity for both heavy oil/water and light oil/water mixtures, and perform with good durability.

Karl Fischer method is usually used to evaluate water content in various organic solvents^54^. The water content in the CCl_4_ after separation was tested by injecting liquid into a titrator for Karl Fischer titration analysis. The water content of CCl_4_ after HHF-PODS separation is ~ 9600 ppm (i.e., ~ 0.96 wt% water). The total organic carbon (TOC) of water after HHF-PODS separation light oil–water mixture (toluene/water) were analyzed by Beiya TOC analyzer (HTY-DI1000C)^55^. The result showed that TOC could be maintained around 9800 ppm. The Karl Fischer and TOC results are well in line with the oil/water separation performance of HHF-PODS.

The abrasion could destroy the surface topography and wettability of HHF-PODS. To evaluate the durability of HHF-PODS, here, the linear abrasion was used to evaluate the durability. Hereby, as showed in Fig. 6f, HHF-PODS was placed on sandpaper (2000 Cw), and a 100 g weight was placed and moved 10 cm with a speed of 2 cm/s^25^. Clearly, HHF-PODS could sustain hydrophobicity and good oil–water separation performance after linear abrasion repeatedly, clarifying the excellent adhesion of the rough PODS coating on the HHF. The evidence of the linear abrasion experiment states clearly that the HHF-PODS is mechanically durable.

## Conclusion

In conclusion, we synthesized HHF-PODS with a facile dip-coating method, inspired by the self-assembly performance and hydrophobicity of OTS modification. The core of our innovation lies in the fact that the remarkable hydrophobic coating rationally surrounded onto the surface of HHF. Notably, the concept was embodied by a rational integration of waste utilization and special wettability, which exhibited extraordinary hydrophobicity and oil/water separation capacity. Moreover, the HHF-PODS can separate oils of different densities by a simple homemade oil/water mixture separation testing system. The results show that the efficiency was satisfactory and all higher than 99.0% for different density oils/water mixtures. In addition, HHF-PODS exhibited a prominent resistance and durability. Benefiting from the merits of superior resistance and durability, this HHF-PODS can withstand harsh environments, maintain outstanding separation ability for both heavy oil/water and light oil/water mixtures, and perform with good durability. Owing to the merits of waste utilization, good hydrophobicity, durability toward different harsh environments, and reusability, the combination of waste material and special wettability may provide a promising avenue for the replacement of traditional oil/water separation materials.

## Supplementary Information

Supplementary Figures.
