# Supplementary material for: Novel fabrication of hydrophobic/oleophilic human hair fiber for efficient oil/water separation through one-pot dip-coating synthesis route
Source: Sci Rep. 2022 May 10;12:7632. doi: 10.1038/s41598-022-11511-2 (PMC9090757; doi:10.1038/s41598-022-11511-2)
Supplement: Supplementary file 1 — Supplementary Figures. [file 41598_2022_11511_MOESM1_ESM.docx]

Supporting Information

**Novel Fabrication of hydrophobic/oleophilic human hair fiber for efficient oil/water separation through one-pot dip-coating synthesis route**

Yang Chenxi ^a, b, c, d^, Wang Jian ^a, b, c, d^, Zhang Haiou ^a, b, c, d^, Cao Tingting ^a, b, c, d^ Zhou hang ^a, b, c, d^, Wang Jiawei ^a, b, c, d^, Bai Bo*^e, f^

a. Institute of Land Engineering and Technology, Shaanxi Provincial Land Engineering Construction Group Co., Ltd., Xi'an 710075, China.

b. ShaanXi Provincial Land Engineering Construction Group Co., Ltd., Xi'an 710075, China.

c. Key Laboratory of Degraded and Unused Land Consolidation Engineering, the Ministry of Natural Resources. Ltd., Xi'an 710075, China.

d. Shaanxi Provincial Land Consolidation Engineering Technology Research Center. Xi'an 710075, China.

e. Key Laboratory of Subsurface Hydrology and Ecological Effects in Arid Region of the Ministry of Education, Chang’an University, No. 126 Yanta Road, Xi’an 710054,

Shanxi, China.

f. School of Water and Environment, Chang’an University, Xi’an 710054, China.


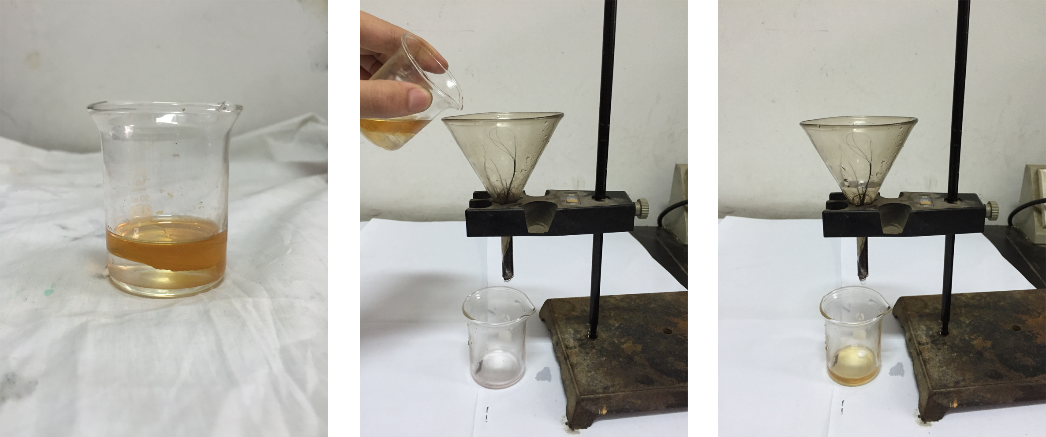


**Figure S1**. Photograph of the oil/water separation by modified HHF-PODS


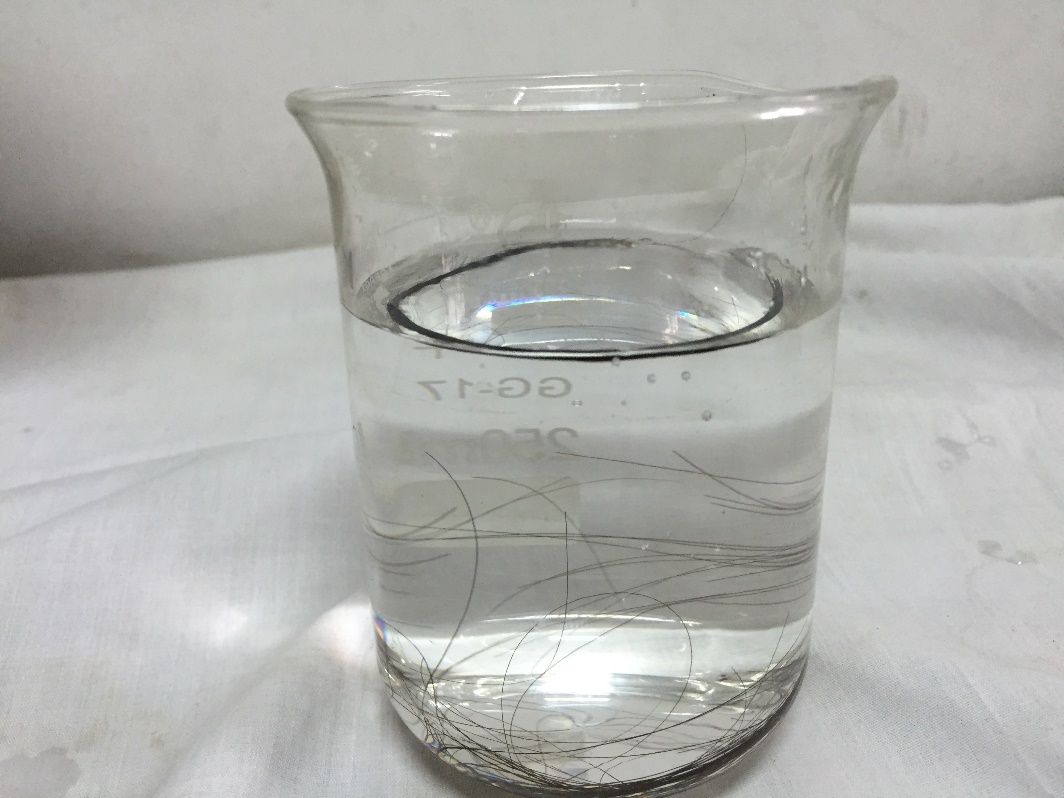


**Figure S2**. The reflections from air bubbles trapped under the water droplet validate the Cassie-Baxter wetting state.
